# Integrated care pilot in north-west London: a mixed methods evaluation

**DOI:** 10.5334/ijic.1149

**Published:** 2013-07-25

**Authors:** Natasha Curry, Matthew Harris, Laura H. Gunn, Yannis Pappas, Ian Blunt, Michael Soljak, Nikolaos Mastellos, Holly Holder, Judith Smith, Azeem Majeed, Agnieszka Ignatowicz, Felix Greaves, Athina Belsi, Nicola Costin-Davis, Jessica D. Jones Nielsen, Geva Greenfield, Elizabeth Cecil, Susan Patterson, Josip Car, Martin Bardsley

**Affiliations:** Health Policy, Nuffield Trust, 59 New Cavendish Street, London, UK; Public Health, Department of Primary Care and Public Health, Imperial College London, UK; Global eHealth Unit & Biostatistician, School of Public Health, Imperial College London, UK; Health Services Research, School of Health Sciences, City University, London, UK; Nuffield Trust, 59 New Cavendish Street, London, UK; Department of Public Health & Primary Care, Imperial College London, UK; Department of Primary Care & Public Health, School of Public Health, Imperial College London, UK; Health Policy, Nuffield Trust, 59 New Cavendish Street, London, UK; Nuffield Trust, 59 New Cavendish Street, London, UK; Department of Primary Care & Public Health, Imperial College London, UK; Department of Primary Care & Public Health, School of Public Health, Imperial College London, UK; Department of Primary Care and Public Health, Imperial College London, UK; Department of Surgery and Cancer, 2nd Floor, Paterson Centre, St Marys Hospital, Imperial College London, UK; Imperial Clinical Research Unit, Imperial College London, UK; Department of Psychology, City University, London, UK; Department of Primary Care & Public Health, Imperial College London, London, UK; Department of Primary Care & Public Health, Imperial College London, UK; Metro North Mental Health, Royal Brisbane and Women's Hospital, Herston, Qld 4029, Australia; Primary Care, Department of Primary Care & Public Health, Imperial College London, UK; Research, Nuffield Trust, London, UK

**Keywords:** integrated health care systems, health services research, health care, reform, patient-centred care, evaluation studies

## Abstract

**Introduction:**

This paper provides the results of a year-long evaluation of a large-scale integrated care pilot in north-west London. The pilot aimed to integrate care across primary, acute, community, mental health and social care for people with diabetes and/or those aged 75+ through care planning, multidisciplinary case reviews, information sharing and project management support.

**Methods:**

The evaluation team conducted qualitative studies of change at organisational, clinician and patient levels (using interviews, focus groups and a survey); and quantitative analysis of change in service use and patient-level clinical outcomes (using patient-level datasets and a matched control study).

**Results:**

The pilot had successfully engaged provider organisations, created a shared strategic vision and established governance structures. However, the engagement of clinicians was variable and there was no evidence to date of significant reductions in emergency admissions. There was some evidence of changes in care processes.

**Conclusion:**

Although the pilot has demonstrated the beginnings of large-scale change, it remains in the early stages and faces significant challenges as it seeks to become sustainable for the longer term. It is critical that National Health Service managers and clinicians have realistic expectations of what can be achieved in a relatively short period of time.

## 1. Introduction

Health care systems in developed countries face similar challenges: increasing numbers of older people, more people living longer with multiple long-term conditions and a need to provide correspondingly complex care against a backdrop of ever-tightening finances. Health and care services are increasingly seeking ways to reduce duplication and drive efficiency without compromising quality [1]. The focus, therefore, has been on how to contain costs by shifting care out of expensive hospital and institutional settings into the community and reducing adverse outcomes such as chronic disease complications or emergency admissions, whilst assuring and ideally improving safety, quality and patient experience. Integration of care is seen as one possible solution to these challenges and is highlighted increasingly by commentators, academics and governments [2] with many countries launching pilots based on the principles of integrated care. Although often perceived as a single organisation, the English National Health Service frequently suffers from fragmentation and lack of coordination between different care organisations [3]. The separation of providers and purchasers of care, along with differing payment systems, means that financial motivations are not naturally aligned across the system.

In view of the multitude of definitions of integrated care, it is important to state that, for the purposes of this paper, our use of the term ‘integrated care’ refers to an approach that seeks to improve the quality of care for individual patients, service users and carers by ensuring that services are well coordinated around their needs [3]. Despite the attractive potential of integrated care, evidence on its effectiveness remains mixed [4]. Some large-scale examples, such as Kaiser and Geisinger in the US, have impressively low rates of emergency admission and readmission [5,6]. One approach evaluated in Torbay in south-west England, which sought to integrate health and social care for older people, has had some success, having reduced emergency bed-day use for people aged 75+ by 24% and by 32% for people aged 85+ between 2003 and 2008 [7]. However, a recent national evaluation of 16 integrated care organisations across England produced equivocal results; whilst there were reductions in planned admissions and in outpatient attendance, there was no evidence of a reduction in emergency admissions. In addition, there was no improvement in patient experience [8]. Results from an earlier evaluation of the US Evercare programme, trialled in England in 2005, are not dissimilar – whilst this programme appeared to score highly in terms of patient satisfaction, it failed to reduce emergency admissions significantly [9].

North-west London is a further example of an integrated care project, although larger in scale than many that have been attempted previously in the English National Health Service. Though there have been some claims for its success [10], these predate the publication of our formal evaluation [11]. Bringing together organisations from primary, secondary, community, mental health and social care sectors, the pilot aims to reduce emergency admissions by providing coordinated, multidisciplinary care to those residents who are aged 75+ and/or living with diabetes. This paper describes the findings of the first year of an evaluation of the north-west London pilot, which launched in July 2011 across a total population of 550,000. Evaluation data was collected between July 2011 and April 2012. This paper seeks to draw out lessons for the implementation and evaluation of other integrated care pilots and reflects on the challenges associated with measuring the impact of, and attributing change to, particular interventions. More detailed information about the set-up of the pilot and our results can be found in the full evaluation report [11].

### 1.1. The north-west London approach

The north-west London integrated care pilot, originally launched for one year in July 2011 but later extended for a further year, constitutes a large-scale change programme involving two hospitals, two mental health providers, three community health care service providers, five municipal providers of social care, two non-governmental organisations and (by the end of April 2012) 103 general practitioners. Its aims are to improve health outcomes and reduce unnecessary admissions to hospital by proactively managing people living with diabetes and/or those aged 75+ through creating better access to more integrated care outside hospital and by enabling effective working of professionals across organisations. The pilot operates as a network – sometimes known as ‘virtual’ integration [12] – with separate provider organisations working together towards common goals according to a set of contractual agreements which are signed upon joining the pilot. Agreements state that providers must operate within a governance structure based on weighted voting rights if the consensus cannot be reached between participating organisations and must share financial savings according to pre-agreed proportions. The pilot's original business case (based on a pilot population of 380,000) projected acute savings of £10.9 m in its first year of operation, rising to £23.2 m by year 5. The pilot was expected to target an estimated 15,200 patients with diabetes and 22,800 patients who are aged 75+; 8700 patients fell into both categories.

**Figure 1.**
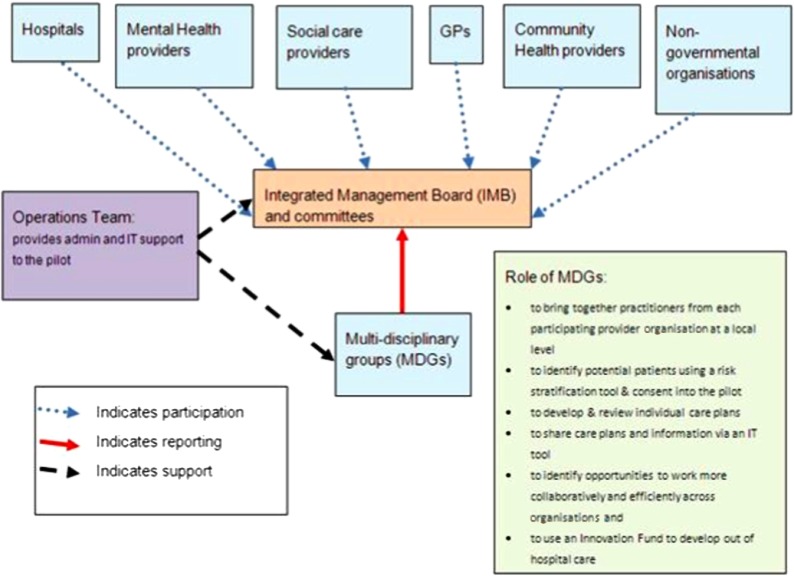
Structure of the north-west London integrated care pilot. Note: GPs, General Practitioners.

The organisational structure of the pilot is set out in Figure 1. Organisations’ participation in the pilot is voluntary and representatives of all organisations involved are invited to attend the monthly Integrated Management Board meetings. The Board is run as a ‘club’ – participating organisations can attend the meetings, or choose not to, but mutual benefits are attained from membership. At a local level, representatives from all provider organisations belong to multidisciplinary groups established to improve care coordination across different services, particularly for patients at high risk of hospitalisation. The representatives are expected to work collaboratively, sharing expertise to improve patient care. General practitioners are expected to create care plans for all patients in the pilot. The care plans were designed by clinicians and are intended to bring standardisation and best practice across the pilot. These are shared amongst participating provider organisations via a bespoke information technology tool, which also displays integrated utilisation data using a data integration platform, and a recent Combined Predictive Model score for hospital admission risk [13]. Organisations are paid to backfill staff time to allow them to attend multidisciplinary group meetings and produce care plans. An Innovation Fund was established so that multidisciplinary group's could commission new community services that support out of hospital care in their respective localities [14]. Members of the multidisciplinary groups decide how they would like to use their allowance (allocated by the size of the group) and submit proposals to the Integrated Management Board for approval. In the first year of the pilot, the Innovation Fund was approximately £450,000 [15].

## 2. Evaluation methods

This was a mixed methods evaluation to assess the implementation and impact of the pilot [16]. We used a mixture of interviews, direct observation and documentary analysis to understand the policy and local contextual factors that shaped the design and implementation of the pilot and to examine the effectiveness of the governance structures and aligned financial incentives. In addition, focus groups and surveys explored the experiences of staff and patients participating in the pilot and examined whether the multidisciplinary groups were successful at improving collaboration and information-sharing between professionals. Table 1 summarises the qualitative data collection. Notes from interviews, observations and focus groups were analysed thematically by some of the research team using a constant comparison [17] within a modified framework approach [18]. Codes were created both horizontally (by coding each interview or focus group as a stand-alone hermeneutic unit) and vertically (by scanning across the data for specific terms), and then developed into categories and themes. Categories were refined and coding reviewed throughout the process for which the Atlas® software was used. Ten hours of multidisciplinary group meeting observation data were transcribed in full, coded and analysed in detail, in a similar method to that described above. The quantitative survey data was analysed using statistical software package SPSS. Ethical and governance approvals were granted by the National Health Service National Research Ethics Service for City and East London.

Between November 2011 and April 2012, the following data were collected:

For the first part of the quantitative component, the impact of the integrated care pilot on service use and cost was assessed using two complementary methods. First, we monitored the service use of the general population of inner north-west London and the pilot's target population by observing patterns of activity in administrative datasets. Eligibility was determined by age and diagnoses on admission to hospital, and represented a rolling cohort of patients. These were contrasted with other areas of London and national datasets. The second part of the analysis examined a fixed cohort of patients who had received a care plan compared to matched individuals taken from other areas with similar population characteristics – this represented changes associated with ‘usual care’. Patterns of hospital use for both groups were compared using a generalised difference-in-differences regression approach at the person level. This approach has been used in a number of earlier studies [19,20]. A wide range of variables were used for matching participants to controls. These were a predictive risk score for emergency hospital admission in the next 12 months: age, sex, prior hospital utilisation, total number of chronic health conditions, area-level deprivation score and history of 15 specific health needs. We assessed the similarity of the matched control group to the group of the pilot's patients by using the standardised difference, where a value greater than 10% is indicative of a meaningful difference between the groups [21]. In both approaches, we tested the level of service utilisation before and after the start point of the pilot or the care plan.

The second component of the quantitative analysis assessed care processes and intermediate health outcomes (e.g. HbA1c control in patients with diabetes and dementia diagnoses in the elderly cohort). We used anonymised patient-level datasets provided by the pilot's Operations Team. Primary care data were available from January 2006 through June 2012, and Secondary Uses Service inpatient data were complete from April 2009 through March 2012. Given that the majority of care plans were completed from January 2012, most patients were exposed to around three months of care planning. Since substantial changes to adverse outcomes such as emergency admissions for chronic diseases are not expected with such a short period of evaluation, we decided it was most relevant to present a range of care process data to provide a broad baseline assessment of performance among the pilot's practices. These include standard *t*-tests and tests for differences in proportions across HbA1c, cholesterol and blood pressure among diabetes patients who had been exposed to at least three months of care planning.

Microsoft Excel and Access were used to manage the data, and Stata (version 11) and SAS (v9.3) for statistical analysis.

## 3. Results: what progress has north-west London made in its first year?

The literature suggests there are a number of key elements that are crucial for successful and effective integrated care [22,23]. The north-west London integrated care pilot was built around these key elements, which include governance structures, financial arrangements, common care processes and an information-sharing platform that enable and encourage collaborative practices underpinned by a shared vision and culture. In establishing these core elements of integrated care, the pilot aimed to improve patient experience and clinical outcomes and to shift care out of the hospital sector. The following section examines the extent to which the pilot was successful in establishing the elements of integrated care and in bringing about its intended impact.

### 3.1. Establishing the structures and engaging organisations and professionals

North-west London's pilot was made possible by project funding of £10 m^1^ from the London Strategic Health Authority (National Health Service London), which enabled investment in governance arrangements, a support team and a data-sharing platform. Other local enablers included a strong central drive from National Health Service London, consistent leadership within the pilot and a shared and pressing need across the local health and social care sectors to achieve ambitious financial savings.

The deliberate decision to run the pilot as a ‘club’ was intended to foster a voluntary and participatory ethos and this has been credited with bringing about high levels of engagement at an organisation level. Furthermore, agreement to share any financial surplus was reported as crucial in overcoming historical tensions and mistrust. Whilst the leadership of organisations demonstrated a high level of engagement and commitment to the pilot, with 40 or more individuals regularly attending the Integrated Management Board meetings, the complex nature of the governance arrangements gave rise to concerns over lines of accountability and clarity of decision-making. Some professionals expressed confusion over financial incentives and over which issues should be escalated to the board. Nearly a third of survey respondents (27.7%) felt that their role and responsibilities within the pilot were unclear. Just over 37% of clinical respondents were confused about the method of retrieving monetary incentives.

We found that the vision and broad principles of integrated care were seen by health and social care professionals to be both timely and a ‘step in the right direction’. However, active engagement amongst clinicians was reported to be variable with 64% reporting that they did not feel they were involved in the planning and development of the pilot. The design and roll out of the information technology tool has proved more complex, time-consuming and costly than anticipated and has given rise to frustration about the level of access to information amongst some (57%) who have tried to use it. Over half of the professionals (56%) were dissatisfied with the degree of integration between the information technology tool and other clinical information systems, in particular the existing electronic patient records systems such as EMIS Web.

The integrated care pilot's ambitious aims were intended in part to drive engagement and to secure support from organisations but concern has been raised amongst some participants that the pilot has in fact been too ambitious, risking disengagement amongst those who may be disappointed that aims around reduced admissions and financial savings have not been achieved in the first year.

### 3.2. Changing care processes

#### 3.2.1. Multidisciplinary groups

Nine multidisciplinary groups were established during the first year of the pilot with a brief to improve care planning for patients aged 75+ or living with diabetes. Eight of the 31 interviewees at a strategic level and 16 of 25 of those at a clinical level reported that the multidisciplinary groups were, by the end of year 1, beginning to generate small changes in practice and even to demonstrate a trend towards a culture of collaborative working. Despite some initial scepticism, the survey revealed that some professionals (57%) reported enjoying the experience of increased face-to-face contact with their colleagues in other organisations and felt that this enhanced inter-professional learning (79%), clinical knowledge (76%) and collaborative working (72%). Others voiced dissatisfaction with the number of meetings and the time commitment and questioned the opportunity cost of their involvement (this was particularly true of single-handed general practitioner).

Whilst the multidisciplinary groups were generally well attended by a range of professionals, communication patterns within the groups were observed to be dominated by either the general practitioners who were delivering a case presentation or the consultants who were providing clinical advice. There was relatively little input from nurses, social care professionals or allied health professionals or from other general practitioners who were participating but not presenting cases. There were significant differences in the proportion of utterances (units of meaning, phrases or sentences expressing a complete thought or significant shift in meaning, object, or subject, identified linguistically or based on intonation [24]) per participant type (consultant, 15%; presenting general practitioner, 39%; Chair, 8%; non-presenting general practitioner, 2%; allied health professional, 5%) (see reference [25] for further details about how an utterance is defined), suggesting that the case discussions might not be truly multidisciplinary. Furthermore, discussions tended to be limited to individual cases and stopped short of debate about how to change the local systems of care. Although there was some evidence that participants developed an improved understanding of the local health economy, only rarely were activities identified to improve ways of working more broadly between the different participating organisations [14,25] and therefore there was little evidence to suggest that the multidisciplinary groups were fostering a significant cultural shift in ways of working. As such, multidisciplinary groups in the first year could be characterised as community-based ward rounds rather than forums to identify efficiency improvements in the local health economy [25].

Accordingly, by the end of the first year of the pilot, the innovation fund remained underspent with only 39% of available funds allocated and few new services established to divert activity from hospital. Use of the innovation fund varied widely between multidisciplinary groups in terms of how and how much was spent. For instance, one multidisciplinary group had spent over £75,000 on a range of services including one to tackle falls whilst two multidisciplinary groups had not allocated any funds.

#### 3.2.2. Care plans

The care planning process was initially slow, partly due to information technology difficulties, but accelerated during the course of the year. By April 2012, the pilot reported that there were 8676 patients with a care plan, 30% of the total possible. The majority had been completed in the three months between January and April 2012. Professionals expressed enthusiasm for the idea of care planning, but also reported dissatisfaction (58%) with the extra time required to create a care plan. They also reported that the pressure to increase the number of plans completed meant that the process had become a ‘tick box’ exercise.

In a survey of 405 completed patient questionnaires, 22% said that they had a care plan and, amongst those, 63% reported that they had been involved in planning their care. The Integrated Management Board scrutinised the performance of multidisciplinary groups in terms of the number of completed care plans but had no clear mechanisms for assessing, or holding groups to account for, their quality. This was the subject of discussions at Integrated Management Board meetings and a need for quality assurance was recognised during the pilot year.

### 3.3. Changes in patient experience and outcomes

#### 3.3.1. Impact on hospitalisations in target populations

The first part of our evaluation of hospitalisations considered patterns of service use for all eligible patients in the pilot practices and four comparator practice groups (all practices in England, the non-pilot practices in inner North-west London, non-pilot practices in outer North-west London and all practices in south-west London). The increase in emergency admission in the pilot was less than that in outer north-west London, but greater than the change at national level and in south-west London (see Table 2). There were different patterns within the eligible population with groups of older people, particularly those without a diagnosis of diabetes, faring generally better than average in terms of a reduction in emergency admissions when compared to all other areas. The groups of people with diabetes (whatever age) tend to have fared worse. Changes in emergency admissions appeared to be linked to the choice of start date and comparator area. When the start date of the pilot is assumed to be September 2011 (the point when practices were ready to begin recruiting patients) there was actually an increase in admissions for all of the pilot's eligible patients relative to the previous year (1.05%, 95% confidence interval: –1.27 to 3.32%), although these changes were not statistically significant.

**Figure 2.**
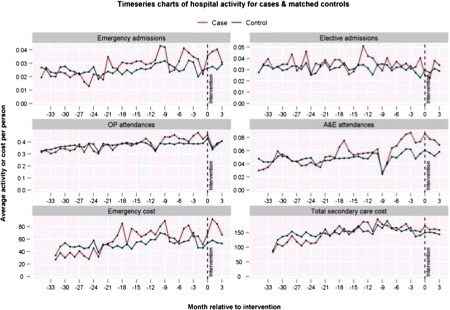
Comparison of patterns of average monthly service use over time for intervention (‘case’) group and their matched controls in inpatient, outpatient and accident and emergency settings.

The second part of our evaluation of hospitalisations considered patterns of service use for a subset of integrated care pilot patients and their matched controls. The pilot recruited 1494 patients for care management before the end of December 2011 (the point at which patients would have at least three months of follow-up data using the most recent datasets available the time the analysis was carried out). Using a number of health variables, the matching process found good matches for 1236 of these patients and created a control group of 5963 patients from areas outside north-west London. The intervention group did not exhibit any significant changes in emergency admission (*p*=0.056), accident and emergency attendances (*p=*0.195), costs of emergency admission (*p=*0.101) or total inpatient costs (*p=*0.871) (Figure 2), nor were there any significant differences between the intervention group and the matched controls with respect to changes in those measures (*p* values ranging from 0.277 to 0.758). The level of emergency admissions in the intervention group appears low for a group targeted specifically for reductions in utilisation. The rate of emergency admission is 0.44 admissions per person per year compared with 0.11 for the general population of the integrated care pilot practices. By comparison, other interventions have been targeted at patients with admission rates 16 times higher than the general population [19].

#### 3.3.2. Patient experience

Patient surveys revealed a high level of support for the pilot. Seventy-eight per cent of survey participants expressed a favourable opinion about the idea of integration of services through better communication between providers from various levels of care. Patients with a care plan expressed enthusiasm towards the process. Two-thirds (65%) felt that they were involved in the design of their care plan in the way they wanted to be and 79% reported that they had a clear understanding of how care planning works. In contrast, respondents who said that they did not have a care plan or were not aware of having one reported very low levels of involvement in the planning of their care (9%).

A high proportion of patient survey respondents felt that they were involved in decision-making about their care (69%). Sixty-two per cent of participants from all patient groups replied that the pilot provided an opportunity to develop a better relationship with their general practitioner and just over half (54%) said it helped them to understand the role of different health and social care professionals in their care. The majority of respondents also felt that the pilot improved inter-professional communication about their care needs (50% and 73% for all respondents and those with a care plan, respectively) and resulted in health care staff asking fewer questions about their medical history (54% and 77%, respectively). Of all patient participants, 58% reported that the pilot enabled easier access to National Health Services and that it has reduced the time that they spent booking appointments (46%). However, despite the general agreement amongst all patient groups on the positive impact of the *process* of the pilot, over half (54%) of the total number of respondents felt that they did not experience any changes at the point of care provision; this may have been due to the fact that most respondents (53.5%) could not recall when or whether they had joined the pilot.

#### 3.3.3. Health outcomes

The evaluation detected improvements in two of the intermediate clinical outcome measures: cholesterol and blood pressure control in people with diabetes. Those exposed to at least three months of the pilot's care planning showed a marginally significant (*p=*0.0472) increase in the percentage of those with good (≤5 mmol/l) cholesterol control (from 80% to 83%), as well as a significant decrease in the average cholesterol reading (from 4.28 to 4.17 mmol/l, *p<*0.0001).

Blood pressure control has shown continual improvement for all diabetes patients in the pilot's practices over the three years between 2009 and 2012. However, with percentages of good control (≤140/80) ranging between 50% and 58% among the pilot's practices throughout 2011–2012, they still lag well behind national prevalence of good blood pressure control (over 80%). Conversely, there was no significant change in the proportion with good blood pressure control prior to and after being on a care plan for at least three months (*p=*0.3809).

**Figure 3.**
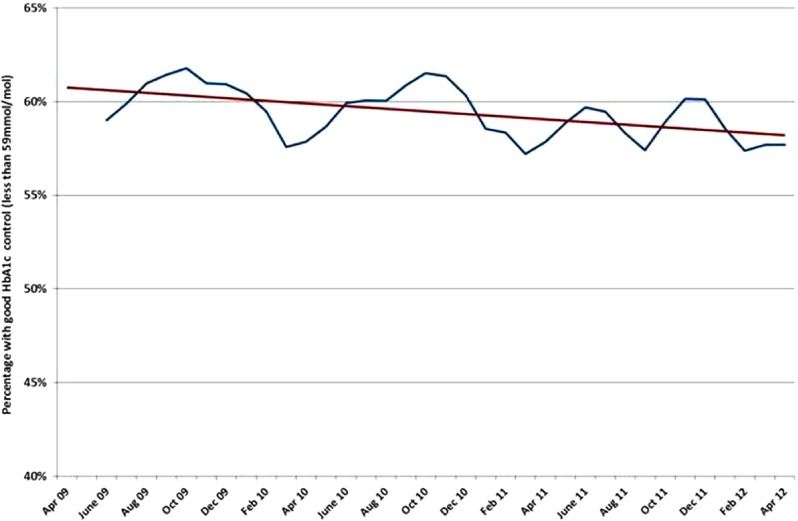
Percentage with good (≤59 mmol/l) HbA1c control by month, three-month rolling average between 2009 and 2012.

In addition, HbA1c control in people with diabetes showed no improvement either in the pilot's practices over the longer term or in patients exposed to the pilot for three months. Figure 3 shows monthly HbA1c data as a smoothed three-month rolling average. Similar to national trends, the proportion of patients meeting the standard is decreasing [6]. In the short evaluation time, there was a significant decrease in the percentage of those with good (≤59 mmol/l) HbA1c control among those who have been on a care plan for at least three months (*p=*0.0001). This paradoxical finding may be due to the more severe patients now being on care plans, and the more severe cases will have poorer control. However, three months, or slightly more, on a care plan is not sufficient time to detect any kind of true effect in one direction or the other.

**Figure 4.**
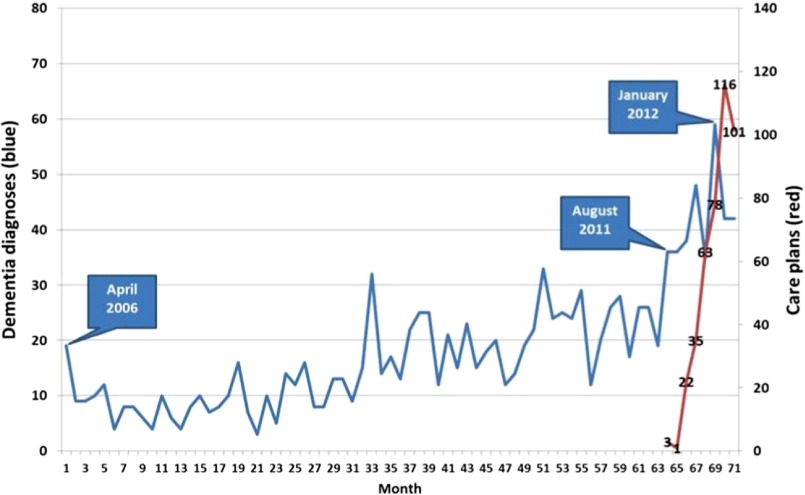
Trends in new cases of practice registered of dementia and care plans for dementia in the integrated care pilot's practices 2006–2012.

Dementia case-finding has become a national priority [26]. For the elderly cohort, Figure 4 shows trends in new cases of practice-registered prevalence of dementia in the pilot's practices between April 2006 and June 2012. There was a rapid increase over the 2011–2012 period of the pilot's start-up. There have been a total of 1353 dementia diagnoses among the pilot's practices since 2006. Interviews with practices registering several new dementia cases revealed that the pilot had an impact as it funded practice nurse time for care planning, and the pilot's care pathway recommended routine cognitive function testing in all patients aged 75+ and at higher risk according to information technology tool. If screening questions were positive then practices performed a 6CIT cognitive function assessment, and if this gave a score <4 patients were referred for blood tests and to a Memory Clinic. Further results using data over a longer period of time, from 2000 to beyond the first year of the pilot will be published in due course.

### 3.4. Summary of results

Table 3 summarises the main findings against the eight key objectives of the pilot, which have been linked to the main elements that the literature suggests are crucial for a successful integrated care organisation. It also reflects on the progress made and challenges remaining.

## 4. Discussion: what can we learn from north-west London?

The north-west London integrated care pilot is a large-scale and ambitious programme of change implemented within a policy context that does not always facilitate collaborative working practices. The evaluation of its first year of operation offers insight into the start-up and early implementation of large-scale change. In its design, the pilot had to overcome inherent tensions within the health and social care system which often mean that different organisations are not necessarily motivated to work together. Establishment of the pilot was enabled by upfront funding and strong central support from the London Strategic Health Authority and consistent leadership across all participating organisations. Our results demonstrate that the pilot has made progress at a strategic level in terms of designing and implementing a complex intervention; in bringing together a large number of diverse provider organisations that span the health and social care sectors; and in creating a common goal and vision to which participating organisations are committed. As such, the pilot has begun to establish the components of integrated care.

However, results of the evaluation also suggest that the pilot remains in the early stages of implementation. Professional experience of the pilot was shown to be mixed, with some signs of early behaviour change as a result of multidisciplinary working but with indications that the vision (so well defined at a strategic level) had not yet been embraced at a clinician or middle manager level. Similarly, the research found some positive indicators of patient experience but the stronger message was that further work was needed to really engage patients, to involve them in care planning and to change their experience of care. Importantly, our analysis of impacts on service use, cost and health outcomes confirms that the pilot's ambitions are yet to be realised. Indeed, reductions in emergency admissions – a central target of the pilot – were not demonstrated during this first year beyond what has been observed elsewhere. Similarly, whilst our analysis detected some early evidence of improvements in diabetes care processes and an increase in dementia case finding, we identified few changes in clinical outcomes. Table 3 summarises the main findings of the evaluation of the first year of the pilot (July 2011–June 2012) and reflects on the main challenges and focus for development in the next phase. This table clearly demonstrates that the pilot has made considerable progress in establishing the structures required for integrated care but that it faces a number of considerable challenges.

The analysis undertaken was contemporaneous with the development of the pilot and so has been able to track change as it happens. One disadvantage is that for the summative elements of this evaluation, it was too early to assess the implications of change in terms of the pilot's intended goals and especially in terms of the impacts on clinical outcomes. One of the major challenges in undertaking this analysis was the length of time over which patients could be followed up. Care planning was slow at first and only accelerated in January 2012. As such, patients’ use of services could only be tracked over a three-month period (January–March 2012) – too short a period to expect a significant change in service use or clinical outcomes. Furthermore, it is also difficult to know how many of the observed organisational changes will endure over longer time periods. Despite the progress that has been made in establishing the pilot, it is probably too early to expect a major impact on service use specifically as a result of the changes in care management and coordination.

Already having secured funding and support to continue for a further year within its existing footprint, the pilot is also set to extend geographically – stretching to encompass outer north-west London. As it moves into its second year, the pilot faces a number of challenges and tensions, some of which are set out in Table 3. A central challenge will be in balancing the need to maintain and increase engagement whilst developing more streamlined decision-making and governance processes. Importantly, the pilot leadership will need to work to embed the vision, which has proved to be strong at a strategic level, throughout all levels of the pilot, paying particular attention to clinicians involved in delivering the care planning and to patients. If significant shifts in the location of care are to be made, the pilot needs to ensure that multidisciplinary groups are given the tools and skills to be able to establish new models of community-based care.

This case study offers weight to the evidence that large-scale change in the National Health Service is complex and takes time [30]. Indeed, many of the models that are providing inspiration to the National Health Service currently (e.g. Kaiser Permanente and Geisinger in the US) have been established for many years, if not decades. As such, it is important to frame this evaluation within the realms of what could realistically be expected within the first year of the pilot. Results from previous large-scale change programmes in the National Health Service point to useful messages for the north-west London integrated care pilot about how long large-scale change takes [9,35–37].

Our evaluation data reveal achievements and challenges that would not be unfamiliar to those who have attempted to bring about large-scale change in the National Health Service in the past. Like these previous studies, this evaluation calls into question whether policy-makers and National Health Service managers expect too much too soon when testing initiatives. Driven by challenging savings targets and an annual financial cycle, National Health Service organisations are under significant pressure to demonstrate the success of initiatives such as the north-west London integrated care pilot. This can lead to overly ambitious aims being established and data being analysed before it is reasonable to expect an impact [10]. Given that the timescales to establish successful integrated care structures can take many years [3,5,7,34], what are reasonable short-term goals for such major changes? How do sponsors of such change gain momentum for a project where intended changes are necessarily long term? Why do many schemes set ambitious short-term targets for such projects to launch, implement and prove their worth? There are no hard and fast answers to these questions but, now that north-west London has secured resources to continue its pilot for a further year and to roll it out rapidly, further evaluation over a longer time period may make it possible to start to detect more of the changes it set out to achieve. Our evaluation points to a number of lessons for managers, clinicians and researchers who are embarking upon a similar development. These are set out below in Box 1.

## 5. Conclusion

This evaluation has contributed to our understanding of the process and mechanisms of establishing a large-scale integrated care project within health and social care in the National Health Service. It has clearly highlighted the challenges and tensions involved in such a pilot and underlined the need for thorough evaluation and research over a long period of time in order to detect impact. This study has provided important learning for others seeking to embark upon an initiative of equivalent scale.

## 6. Reviewers

**Caroline Dickinson**, Director, Mater/UQ Centre for Primary Health Care Innovation, Mater Health Services, Queensland, Australia

**Alexander Pimperl**, Head of Healthcare Analytics/Controlling & IT Department, OptiMedis AG, Hamburg, Germany

**Lara Sonola**, Senior Researcher, The King's Fund, London, UK

## Figures and Tables

**Box 1.**
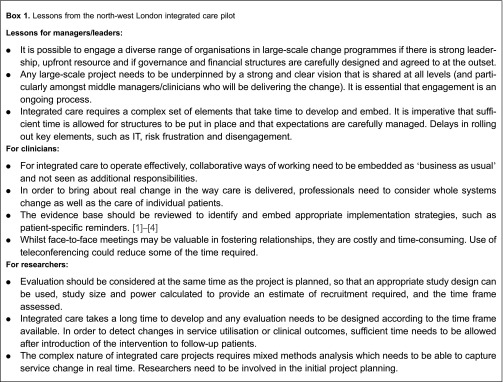
Lessons from the north-west London integrated care pilot

**Table 1.**
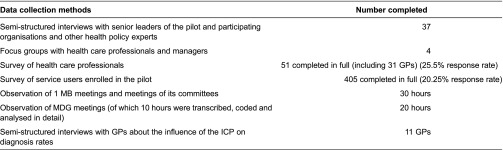
Summary of qualitative methods

**Table 2.**
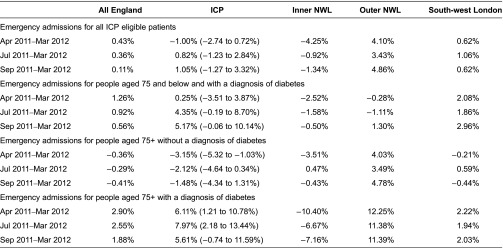
Changes over time in number of emergency admissions, by eligibility type, for integrated care pilot practices and the four comparator practice groups. Pilot results have 95% confidence levels.

Note: NWL, north-west London

**Table 3.**
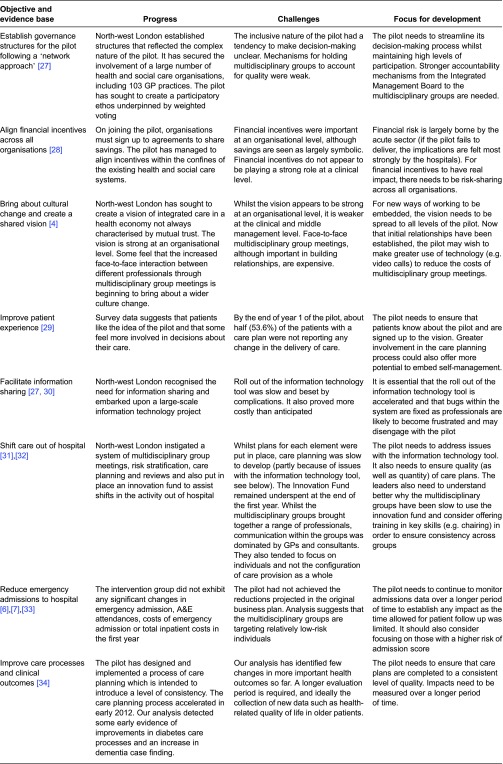
Summary results

## Footnotes

1 Under changes to the emergency admissions tariff introduced in 2010, local commissioners pay 100% of the tariff for an emergency admission but the trust retains only 30% for emergency admissions above 2008/2009 levels; the remaining 70% goes to National Health Service London. National Health Service London, following negotiations with North-west London PCTs, agreed to release this money back into the sector to fund the pilot.
